# Supplementary material for: A Simulation Study of the Ecological Speciation Conditions in the Galician Marine Snail Littorina saxatilis
Source: Front Genet. 2022 Apr 5;13:680792. doi: 10.3389/fgene.2022.680792 (PMC9037070; doi:10.3389/fgene.2022.680792)
Supplement: Supplementary file 1 [file DataSheet2.PDF]

A simulation study of the ecological speciation conditions in the Galician marine snail *Littorina saxatilis*

M. Fernández-Meirama[1], E. Rolán-Alvarez[1], A. Carvajal-Rodríguez[1*]

[1] Departamento de Bioquímica, Genética e Inmunología and Centro de Investigación Mariña (CIM), Universidade de Vigo, Vigo, Spain

* **Correspondence:**

Corresponding Author

acraaj@uvigo.es

Keywords: ***L. saxatilis*, assortative mating, ecological speciation, mate choice evolution, sympatric speciation**

Table S1. Model parameter values for the comparison between Galician model and a symmetric model regarding carrying capacity and migration.

			Value	
	**Parameter**	**Symbol**	**Galician**	**Symmetric**
Demography	Generation number	T	20,000	20,000
	**Number of exposed demes**		**4**	**12**
	Number of intermediate demes		2	2
	**Number of sheltered demes**		**20**	**12**
	**Per deme carrying capacity in exposed habitat**	K	**15,000**	**5481**
	**Per deme carrying capacity in intermediate and sheltered**		**3750**	**5481**
	Mean offspring number	b	50	50
	Between deme migration probability	m		
		0 demes	0.75	0.75
		1 demes	0.15	0.15
		2 demes	0.1	0.1
Genome Structure	Number of microsatellites		L = 8	L = 8
	Ecological magic trait (*L* loci)	x	L = 4	L = 4
	Male mating trait (*L* loci)	c	L = 4	L = 4
Selection	Selection strength	σ_s	0.45	0.45
	Habitat optimum selection in middle	θ	no (neutral)	no (neutral)
Mating	Evaluated females per male	N_f	10	10
	Mating preference tolerance	σ_a	0.05	0.05
	Mate choice cost		no	no
Mutation	Neutral mutation rate per locus	μ_0	10^{-3}	10^{-3}
	Trait mutation rate per locus	μ	10^{-5}	10^{-5}

Table S2. Results for the ecological trait and choosiness under Galician and Symmetric models for the parameters given in Table S1 i.e. *L*= 4 loci, intermediate selection strength σ_s = 0.45, and tolerance σ_a = 0.05 and neutral model in the middle habitat. Values are averages of 100 replicates plus minus the standard error of the mean.

Model	x trait value in lower shore	x trait value in middle shore	Choosiness (*C*)		
			Upper	Middle	Lower
Galician	0.0005± 0.0002	0.15 ± 0.004	0.52±0.019	0.54±0.016	0.54 ±0.018
Symmetric	0.007 ± 0.0001	0.48 ± 0.007	0.58±0.018	0.58±0.015	0.56±0.015

Table S3. Cases with evolution of negative assortative mating defined as *C*<-0.1 (c<0.45). Values are computed over 20 replicates. Empty cells imply no colonization of the lower shore.

σ_s	σ_a	Middle zone	*L*	% runs with negative *C*			Mean *C*		
				Lower	Middle	Upper	Lower	Middle	Upper
0.15	0.1	selective	4	65%	75%	80%	-0.32	-0.36	-0.38
0.15	0.1	neutral	4	50%	50%	55%	-0.21	-0.07	-0.12
0.15	0.05	selective	4	90%	95%	95%	-0.5	-0.55	-0.51
0.15	0.05	neutral	4	80%	80%	95%	-0.27	-0.26	-0.42
0.15	0.1	selective	8	15%	25%	35%	0.08	0.08	-0.02
0.15	0.1	neutral	8	---	25%	20%	---	0.04	0.05
0.15	0.05	selective	8	0%	0%	5%	0.1	0.18	0.1
0.15	0.05	neutral	8	---	5%	0%	---	0.14	0.14

Table S4. Results for the Galician model for the parameters given in Table S1 i.e. *L*= 4 loci, intermediate selection strength σ_s = 0.45, and tolerance σ_a = 0.05 but comparing the choosiness in the two middle habitat scenarios i.e. neutral versus selective. Values are averages of 100 replicates plus minus the standard error of the mean.

Middle Scenario	Choosiness (*C*)		
	Upper	Middle	Lower
Neutral	0.52±0.02	0.54±0.02	0.54 ±0.02
Selective (θ=0.5)	0.52±0.02	0.53±0.02	0.53±0.02

Table S5. Results for the Galician model for the same parameters as in Table S4 but comparing the mating correlation instead of choosiness. Values are averages of 100 replicates plus minus the standard error of the mean.

**Middle Scenario**	**Correlation (*r*)**		
	**Upper**	**Middle**	**Lower**
Neutral	0.63±0.04	0.85±0.01	0.79 ±0.04
Selective (θ=0.5)	0.63±0.04	0.87±0.02	0.69±0.04

Table S6. Results for mating correlation under the Galician and the Symmetric model for the parameters given in Table S1 i.e. *L*= 4 loci, intermediate selection strength σ_s = 0.45, and tolerance σ_a = 0.05 and neutral model in the middle habitat. Values are averages of 100 replicates plus minus the standard error of the mean.

**Model**	**Correlation (*r*)**		
	**Upper**	**Middle**	**Lower**
Galician	0.63±0.04	0.85±0.01	0.79 ±0.04
Symmetric	0.73±0.04	0.99±0.002	0.81±0.04

Table S7. Percentage of speciation, defined as mate choice trait c ≥ 0.9 in the middle habitat and Q_{ST} ≥ 0.9, for the scenarios with high (σ_s =0.15), intermediate (σ_s =0.45) or low selection (σ_s =1) under neutral or selective middle habitat. Tolerance was σ_a = 0.05. *L* is the number of loci for the ecological and male mating traits. Values are averages of 100 replicates.

**σ_s**	**Middle zone**	***L***	**% speciation**
0.15	neutral	4	0%
0.15	neutral	16	0%
0.15	selective	4	0%
0.15	selective	16	0%
0.45	neutral	4	11%
0.45	neutral	16	0%

0.45	selective	4	11%
0.45	selective	16	0%
1	neutral	4	15%
1	neutral	16	0%
1	selective	4	13%
1	selective	16	0%

Table S8. ANOVA results for the scenarios with mating cost, showing % of variance explained by each factor for the ecological (*x*) and choice (*c*) traits, mating correlation (*r*), population size (*N*) and differentiation measures (F_{ST} and Q_{ST}). Only the two most important factor interactions are presented. The values within cells correspond to the % at lower, middle, upper shore, by this order, except for differentiation. The asterisk indicates significance at the 0.001 level.

**Factor**	**Response**					
	x	***c***	***r***	***N***	F_{ST}	Q_{ST}
σ_s	67*, 27*, 74*	39, 45, 47*	12, 85*, 87*	29, 20*, 23	65*	14*
σ_a	0, 0, 3	0, 1, 0	11, 2, 0	6, 0, 0	3	0
**L**	11*, 11*, 2	10, 3, 0	1, 4, 0	7, 7*, 7	6*	14*
θ	0, 8*, 3	24, 6, 2	17, 1, 0	1, 23*, 1	1	14*
L× σ_s	10, 14*, 2	4, 8, 12	8, 1, 4	1, 10*, 7	4*	14*
$\theta \times \sigma_s$	0, 15*, 5	1, 10, 8	2, 0, 1	0, 19*, 7	7*	14*
