# Supplementary material for: A Simulation Study of the Ecological Speciation Conditions in the Galician Marine Snail Littorina saxatilis
Source: Front Genet. 2022 Apr 5;13:680792. doi: 10.3389/fgene.2022.680792 (PMC9037070; doi:10.3389/fgene.2022.680792)
Supplement: Supplementary file 2 [file DataSheet1.PDF]

Unifactorial ANOVA for the Table 3:

Below we present the factorial ANOVA (all factors fixed) for the different dependent variables and the four dependent variables analysed: σ_s: Selection strength, σ_a: Mating strength, L: Number of loci, θ: Selection in the mid-shore. A summary with the most important factors and interactions are presented in Table 3. Notice that we present the relative (%) partial eta in relation to the eta total sum in order to compare across tables.

Ecological trait x. Lower habitat.

Tests of Between-Subjects Effects[a]

Dependent Variable:

Source	Type III Sum of Squares	df	Mean Square	F	Sig.	Partial Eta Squared	% Eta
Θ	0.004	1	0.004	3.986	0.047	0.010	0.04
L	0.000	1	0.000	0.222	0.638	0.001	0.00
σ_a	0.003	1	0.003	2.948	0.087	0.007	0.03
σ_s	0.042	2	0.021	21.607	0.000	0.097	0.40
Θ * L	0.002	1	0.002	2.072	0.151	0.005	0.02
Θ * σ_a	0.000	1	0.000	0.271	0.603	0.001	0.00
Θ * σ_s	0.001	2	0.001	0.757	0.470	0.004	0.02
L * σ_a	0.002	1	0.002	2.418	0.121	0.006	0.02
L * σ_s	0.045	2	0.022	22.942	0.000	0.103	0.42
σ_a * σ_s	0.000	2	0.000	0.214	0.808	0.001	0.00
Θ * L * σ_a	0.001	1	0.001	0.786	0.376	0.002	0.01
Θ * L * σ_s	0.000	1	0.000	0.118	0.732	0.000	0.00
Θ * σ_a * σ_s	0.002	2	0.001	0.917	0.401	0.005	0.02
L * σ_a * σ_s	0.001	2	0.000	0.448	0.639	0.002	0.01
Θ * L * σ_a * σ_s	0.001	1	0.001	0.532	0.466	0.001	0.01
Error	0.390	401	0.001				
Total	0.688	423					
Corrected Total	0.513	422					

a. habitatControl = Lower

b. R Squared = 0.240 (Adjusted R Squared = 0.200)

Ecological trait x. Middle habitat.

Tests of Between-Subjects Effects[a]

Dependent Variable:

Source	Type III Sum of Squares	df	Mean Square	F	Sig.	Partial Eta Squared	% Eta
Θ	0.356	1	0.356	39.320	0.000	0.080	0.03
L	1.039	1	1.039	114.728	0.000	0.203	0.09
σ_a	0.090	1	0.090	9.925	0.002	0.022	0.01
σ_s	18.232	2	9.116	1006.99<br>2	0.000	0.817	 0.35
Θ * L	0.627	1	0.627	69.261	0.000	0.133	0.06
Θ * σ_a	0.056	1	0.056	6.139	0.014	0.013	0.01
Θ * σ_s	1.991	2	0.995	109.955	0.000	0.328	0.14

L * σ_a	0.111	1	0.111	12.212	0.001	0.026	0.01
L * σ_s	1.893	2	0.946	104.552	0.000	0.317	0.13
σ_a * σ_s	0.148	2	0.074	8.168	0.000	0.035	0.01
ϴ * L * σ_a	0.038	1	0.038	4.224	0.040	0.009	0.00
ϴ * L * σ_s	1.403	2	0.701	77.475	0.000	0.256	0.11
ϴ * σ_a * σ_s	0.165	2	0.082	9.109	0.000	0.039	0.02
L * σ_a * σs	0.144	2	0.072	7.967	0.000	0.034	0.01
ϴ * L * σ_a * σ_s	0.175	2	0.087	9.650	0.000	0.041	0.02
Error	4.083	451	0.009				
Total	85.551	475					
Corrected Total	30.986	474					

a. habitatControl = Middle
b. R Squared = 0.868 (Adjusted R Squared = 0.862)

Ecological trait x. Upper habitat.

Tests of Between-Subjects Effects[a]

Dependent Variable:

Source	Type III Sum of Squares	df	Mean Square	F	Sig.	Partial Eta Squared	% Eta
ϴ	0.001	1	0.001	3.258	0.072	0.007	0.01
L	0.000	1	0.000	0.256	0.613	0.001	0.00
σ_a	0.020	1	0.020	43.871	0.000	0.088	0.09
σ_s	0.066	2	0.033	72.542	0.000	0.241	0.26
ϴ * L	1.554E-07	1	1.554E-07	0.000	0.985	0.000	0.00
ϴ * σ_a	0.001	1	0.001	3.210	0.074	0.007	0.01
ϴ * σ_s	1.900E-05	2	9.502E-06	0.021	0.979	0.000	0.00
L * σ_a	0.015	1	0.015	34.104	0.000	0.070	0.07
L * σ_s	0.086	2	0.043	95.189	0.000	0.295	0.31
σ_a * σ_s	0.047	2	0.023	51.630	0.000	0.185	0.20
ϴ * L * σ_a	3.454E-05	1	3.454E-05	0.076	0.782	0.000	0.00
ϴ * L * σ_s	0.000	2	0.000	0.366	0.694	0.002	0.00
ϴ * σ_a * σ_s	0.000	2	0.000	0.293	0.746	0.001	0.00
L * σ_a * σ_s	0.009	2	0.004	9.783	0.000	0.041	0.04
ϴ * L * σ_a * σ_s	0.000	2	7.856E-05	0.174	0.840	0.001	0.00
Error	0.206	456	0.000				
Total	460.310	480					
Corrected Total	0.452	479					

a. habitatControl = Upper
b. R Squared = 0.544 (Adjusted R Squared = 0.521)

Mate choice trait c. Lower habitat.

Tests of Between-Subjects Effects[a]

Dependent Variable:

Source	Type III Sum of Squares	df	Mean Square	F	Sig.	Partial Eta Squared	% Eta
ϴ	37991.076	1	37991.076	150.400	0.000	0.248	0.12

L	28247.154	1	28247.154	111.826	0.000	0.197	0.09
σ_a	1695.565	1	1695.565	6.712	0.010	0.015	0.01
σ_s	136167.732	2	68083.866	269.532	0.000	0.542	0.26
Θ * L	10998.674	1	10998.674	43.542	0.000	0.087	0.04
Θ * σ_a	1013.813	1	1013.813	4.014	0.046	0.009	0.00
Θ * σ_s	76337.454	2	38168.727	151.103	0.000	0.399	0.19
L * σ_a	1002.562	1	1002.562	3.969	0.047	0.009	0.00
L * σ_s	55775.814	2	27887.907	110.403	0.000	0.326	0.16
σ_a * σ_s	3292.227	2	1646.113	6.517	0.002	0.028	0.01
Θ * L * σ_a	1676.684	1	1676.684	6.638	0.010	0.014	0.01
Θ * L * σ_s	21882.385	2	10941.192	43.314	0.000	0.160	0.08
Θ * σ_a * σ_s	2027.427	2	1013.714	4.013	0.019	0.017	0.01
L * σ_a * σ_s	2007.760	2	1003.880	3.974	0.019	0.017	0.01
Θ * L * σ_a * σ_s	3350.627	2	1675.314	6.632	0.001	0.028	0.01
Error	115185.655	456	252.600				
Total	558842.947	480					
Corrected Total	498652.607	479					

a. habitatControl = Lower
b. R Squared = 0.769 (Adjusted R Squared = 0.757)

Mate choice trait c. Middle habitat.

Tests of Between-Subjects Effects[a]

Dependent Variable:

Source	Type III Sum of Squares	df	Mean Square	F	Sig.	Partial Eta Squared	% Eta
Θ	539.857	1	539.857	5.707	0.017	0.012	0.09
L	513.838	1	513.838	5.432	0.020	0.012	0.09
σ_a	23.515	1	23.515	0.249	0.618	0.001	0.00
σ_s	1211.617	2	605.809	6.404	0.002	0.027	0.20
Θ * L	494.139	1	494.139	5.224	0.023	0.011	0.08
Θ * σ_a	20.705	1	20.705	0.219	0.640	0.000	0.00
Θ * σ_s	1037.898	2	518.949	5.486	0.004	0.023	0.17
L * σ_a	23.411	1	23.411	0.247	0.619	0.001	0.00
L * σ_s	930.535	2	465.267	4.919	0.008	0.021	0.16
σ_a * σ_s	36.275	2	18.138	0.192	0.826	0.001	0.01
Θ * L * σ_a	21.003	1	21.003	0.222	0.638	0.000	0.00
Θ * L * σ_s	1011.831	2	505.916	5.348	0.005	0.023	0.17
Θ * σ_a * σ_s	41.324	2	20.662	0.218	0.804	0.001	0.01
L * σ_a * σ_s	45.834	2	22.917	0.242	0.785	0.001	0.01
Θ * L * σ_a * σ_s	40.882	2	20.441	0.216	0.806	0.001	0.01
Error	43134.355	456	94.593				
Total	49208.529	480					
Corrected Total	49127.019	479					

a. habitatControl = Middle
b. R Squared = 0.122 (Adjusted R Squared = 0.078)

Mate choice trait c. Upper habitat.

Tests of Between-Subjects Effects[a]

Dependent Variable:

Source	Type III	df	Mean	F	Sig.	Partial Eta	% Eta

	Sum of Squares		Square			Squared	
Θ	0.188	1	0.188	12.221	0.001	0.026	0.03
L	1.062E-05	1	1.062E-05	0.001	0.979	0.000	0.00
σ_a	0.049	1	0.049	3.161	0.076	0.007	0.01
σ_s	8.634	2	4.317	280.159	0.000	0.551	0.58
Θ * L	0.053	1	0.053	3.466	0.063	0.008	0.01
Θ * σ_a	0.033	1	0.033	2.133	0.145	0.005	0.00
Θ * σ_s	0.033	2	0.016	1.062	0.347	0.005	0.00
L * σ_a	0.145	1	0.145	9.389	0.002	0.020	0.02
L * σ_s	2.743	2	1.372	89.017	0.000	0.281	0.30
σ_a * σ_s	0.179	2	0.090	5.812	0.003	0.025	0.03
Θ * L * σ_a	1.376E-07	1	1.376E-07	0.000	0.998	0.000	0.00
Θ * L * σ_s	0.005	2	0.003	0.165	0.848	0.001	0.00
Θ * σ_a * σ_s	0.010	2	0.005	0.338	0.714	0.001	0.00
L * σ_a * σ_s	0.124	2	0.062	4.016	0.019	0.017	0.02
Θ * L * σ_a * σ_s	0.020	2	0.010	0.659	0.518	0.003	0.00
Error	7.027	456	0.015				
Total	201.767	480					
Corrected Total	19.243	479					

a. habitatControl = Upper
b. R Squared = 0.635 (Adjusted R Squared = 0.616)

Pearson correlation r. Lower habitat.

Tests of Between-Subjects Effects

Dependent Variable:

Source	Type III Sum of Squares	df	Mean Square	F	Sig.	Partial Eta Squared	% Eta
Θ	0.323	1	0.323	9.837	0.002	0.030	0.02
L	1.314	1	1.314	40.090	0.000	0.112	0.08
σ_a	0.852	1	0.852	25.995	0.000	0.076	0.05
σ_s	20.040	2	10.020	305.604	0.000	0.658	0.46
Θ * L	0.007	1	0.007	0.203	0.653	0.001	0.00
Θ * σ_a	0.002	1	0.002	0.068	0.794	0.000	0.00
Θ * σ_s	0.334	2	0.167	5.095	0.007	0.031	0.02
L * σ_a	0.153	1	0.153	4.657	0.032	0.014	0.01
L * σ_s	8.959	2	4.480	136.628	0.000	0.463	0.32
σ_a * σ_s	0.332	2	0.166	5.056	0.007	0.031	0.02
Θ * L * σ_a	0.000	1	0.000	0.004	0.952	0.000	0.00
Θ * L * σ_s	0.023	1	0.023	0.715	0.398	0.002	0.00
Θ * σ_a * σ_s	0.137	2	0.069	2.090	0.125	0.013	0.01
L * σ_a * σ_s	0.034	2	0.017	0.522	0.594	0.003	0.00
Θ * L * σ_a * σ_s	0.001	1	0.001	0.027	0.870	0.000	0.00
Error	10.394	317	0.033				
Total	56.158	339					
Corrected Total	54.103	338					

a. R Squared = 0.808 (Adjusted R Squared = 0.795)

Pearson correlation r. Middle habitat.

Tests of Between-Subjects Effects

Dependent Variable:

Source	Type III Sum of Squares	df	Mean Square	F	Sig.	Partial Eta Squared	% Eta
Θ	0.118	1	0.118	2.482	0.116	0.006	0.00
L	0.183	1	0.183	3.858	0.050	0.009	0.01
σ_a	2.365	1	2.365	49.754	0.000	0.109	0.09
σ_s	82.472	2	41.236	867.503	0.000	0.810	0.64
Θ * L	0.013	1	0.013	0.265	0.607	0.001	0.00
Θ * σ_a	0.133	1	0.133	2.793	0.095	0.007	0.01
Θ * σ_s	0.606	2	0.303	6.379	0.002	0.030	0.02
L * σ_a	0.437	1	0.437	9.200	0.003	0.022	0.02
L * σ_s	4.612	2	2.306	48.512	0.000	0.192	0.15
σ_a * σ_s	0.952	2	0.476	10.009	0.000	0.047	0.04
Θ * L * σ_a	0.032	1	0.032	0.670	0.414	0.002	0.00
Θ * L * σ_s	0.068	2	0.034	0.711	0.492	0.003	0.00
Θ * σ_a * σ_s	0.084	2	0.042	0.889	0.412	0.004	0.00
L * σ_a * σ_s	0.137	2	0.069	1.444	0.237	0.007	0.01
Θ * L * σ_a * σ_s	0.130	2	0.065	1.363	0.257	0.007	0.01
Error	19.346	407	0.048				
Total	235.733	431					
Corrected Total	126.459	430					

a. R Squared = 0.847 (Adjusted R Squared = 0.838)

Pearson correlation r. Upper habitat.

Tests of Between-Subjects Effects

Dependent Variable:

Source	Type III Sum of Squares	df	Mean Square	F	Sig.	Partial Eta Squared	% Eta
Θ	0.067	1	0.067	2.234	0.136	0.006	0.00
L	6.554	1	6.554	217.102	0.000	0.356	0.18
σ_a	0.056	1	0.056	1.844	0.175	0.005	0.00
σ_s	104.745	2	52.373	1734.934	0.000	0.898	0.45
Θ * L	0.007	1	0.007	0.216	0.642	0.001	0.00
Θ * σ_a	0.123	1	0.123	4.066	0.044	0.010	0.01
Θ * σ_s	0.023	2	0.011	0.380	0.684	0.002	0.00
L * σ_a	0.059	1	0.059	1.951	0.163	0.005	0.00
L * σ_s	17.726	2	8.863	293.606	0.000	0.600	0.30
σ_a * σ_s	0.454	2	0.227	7.521	0.001	0.037	0.02
Θ * L * σ_a	0.012	1	0.012	0.386	0.535	0.001	0.00
Θ * L * σ_s	0.002	2	0.001	0.034	0.966	0.000	0.00
Θ * σ_a * σ_s	0.096	2	0.048	1.596	0.204	0.008	0.00
L * σ_a * σ_s	0.684	2	0.342	11.330	0.000	0.055	0.03
Θ * L * σ_a * σ_s	0.071	2	0.035	1.169	0.312	0.006	0.00
Error	11.833	392	0.030				
Total	175.122	416					
Corrected Total	152.566	415					

a. R Squared = 0.922 (Adjusted R Squared = 0.918)

Population size N. Lower habitat.

Tests of Between-Subjects Effects

Dependent Variable:

Source	Type III Sum of Squares	df	Mean Square	F	Sig.	Partial Eta Squared	% Eta
ϴ	1783762.752	1	1783762.752	158.959	0.000	0.258	0.12
L	902980.752	1	902980.752	80.468	0.000	0.150	0.07
σ_a	55846.888	1	55846.888	4.977	0.026	0.011	0.01
σ_s	8459983.308	2	4229991.654	376.952	0.000	0.623	0.30
ϴ * L	544895.326	1	544895.326	48.558	0.000	0.096	0.05
ϴ * σ_a	59385.252	1	59385.252	5.292	0.022	0.011	0.01
ϴ * σ_s	3628669.419	2	1814334.710	161.683	0.000	0.415	0.20
L * σ_a	29610.208	1	29610.208	2.639	0.105	0.006	0.00
L * σ_s	1800920.900	2	900460.450	80.244	0.000	0.260	0.12
σ_a * σ_s	108253.347	2	54126.674	4.823	0.008	0.021	0.01
ϴ * L * σ_a	97256.367	1	97256.367	8.667	0.003	0.019	0.01
ϴ * L * σ_s	1099830.689	2	549915.345	49.005	0.000	0.177	0.08
ϴ * σ_a * σ_s	98981.302	2	49490.651	4.410	0.013	0.019	0.01
L * σ_a * σ_s	64589.889	2	32294.945	2.878	0.057	0.012	0.01
ϴ * L * σ_a * σ_s	149632.599	2	74816.300	6.667	0.001	0.028	0.01
Error	5117027.912	456	11221.552				
Total	145947955.625	480					
Corrected Total	24001626.912	479					

a. R Squared = 0.787 (Adjusted R Squared = 0.776)

Population size N. Middle habitat.

Tests of Between-Subjects Effects

Dependent Variable:

Source	Type III Sum of Squares	df	Mean Square	F	Sig.	Partial Eta Squared	% Eta
ϴ	374292.399	1	374292.399	3519.883	0.000	0.886	0.34
L	1712.994	1	1712.994	16.109	0.000	0.034	0.01
σ_a	520.103	1	520.103	4.891	0.027	0.011	0.00
σ_s	139712.658	2	69856.329	656.936	0.000	0.744	0.29
ϴ * L	3708.629	1	3708.629	34.876	0.000	0.072	0.03
ϴ * σ_a	84.530	1	84.530	0.795	0.373	0.002	0.00
ϴ * σ_s	121693.145	2	60846.572	572.207	0.000	0.717	0.28
L * σ_a	112.114	1	112.114	1.054	0.305	0.002	0.00
L * σ_s	2122.971	2	1061.485	9.982	0.000	0.042	0.02
σ_a * σ_s	500.024	2	250.012	2.351	0.096	0.010	0.00
ϴ * L * σ_a	108.304	1	108.304	1.018	0.313	0.002	0.00
ϴ * L * σ_s	2765.206	2	1382.603	13.002	0.000	0.055	0.02
ϴ * σ_a * σ_s	321.299	2	160.649	1.511	0.222	0.007	0.00
L * σ_a * σ_s	296.776	2	148.388	1.395	0.249	0.006	0.00
ϴ * L * σ_a * σ_s	158.283	2	79.141	0.744	0.476	0.003	0.00
Error	47957.813	451	106.337				
Total	7854326.500	475					
Corrected Total	688700.645	474					

a. R Squared = 0.930 (Adjusted R Squared = 0.927)

Population size N. Upper habitat.

Tests of Between-Subjects Effects

Dependent Variable:

Source	Type III Sum of Squares	df	Mean Square	F	Sig.	Partial Eta Squared	% Eta
ϴ	9.394	1	9.394	0.505	0.478	0.001	0.00
L	3901.365	1	3901.365	209.874	0.000	0.315	0.21
σ_a	102.536	1	102.536	5.516	0.019	0.012	0.01
σ_s	9249.219	2	4624.610	248.780	0.000	0.522	0.35
ϴ * L	6.924	1	6.924	0.372	0.542	0.001	0.00
ϴ * σ_a	0.854	1	0.854	0.046	0.830	0.000	0.00
ϴ * σ_s	209.113	2	104.556	5.625	0.004	0.024	0.02
L * σ_a	143.500	1	143.500	7.720	0.006	0.017	0.01
L * σ_s	8213.575	2	4106.787	220.924	0.000	0.492	0.33
σ_a * σ_s	344.358	2	172.179	9.262	0.000	0.039	0.03
ϴ * L * σ_a	5.208E-06	1	5.208E-06	0.000	1.000	0.000	0.00
ϴ * L * σ_s	58.007	2	29.003	1.560	0.211	0.007	0.00
ϴ * σ_a * σ_s	119.117	2	59.559	3.204	0.042	0.014	0.01
L * σ_a * σ_s	253.377	2	126.688	6.815	0.001	0.029	0.02
ϴ * L * σ_a * σ_s	51.659	2	25.829	1.389	0.250	0.006	0.00
Error	8476.639	456	18.589				
Total	10237475.153	480					
Corrected Total	31139.638	479					

a. R Squared = 0.728 (Adjusted R Squared = 0.714)

Fst.

Tests of Between-Subjects Effects

Dependent Variable:

Source	Type III Sum of Squares	df	Mean Square	F	Sig.	Partial Eta Squared	% Eta
ϴ	0.000	1	0.000	0.665	0.415	0.001	0.00
L	0.030	1	0.030	44.664	0.000	0.089	0.07
σ_a	0.010	1	0.010	14.966	0.000	0.032	0.02
σ_s	0.487	2	0.244	366.366	0.000	0.616	0.48
ϴ * L	0.001	1	0.001	1.543	0.215	0.003	0.00
ϴ * σ_a	0.000	1	0.000	0.689	0.407	0.002	0.00
ϴ * σ_s	0.002	2	0.001	1.236	0.292	0.005	0.00
L * σ_a	0.007	1	0.007	10.986	0.001	0.024	0.02
L * σ_s	0.199	2	0.100	149.990	0.000	0.397	0.31
σ_a * σ_s	0.025	2	0.012	18.498	0.000	0.075	0.06
ϴ * L * σ_a	0.000	1	0.000	0.235	0.628	0.001	0.00
ϴ * L * σ_s	0.003	2	0.002	2.344	0.097	0.010	0.01
ϴ * σ_a * σ_s	0.002	2	0.001	1.336	0.264	0.006	0.00
L * σ_a * σ_s	0.003	2	0.002	2.547	0.079	0.011	0.01
ϴ * L * σ_a * σ_s	0.000	2	9.180E-05	0.138	0.871	0.001	0.00
Error	0.303	456	0.001				
Total	5.811	480					
Corrected Total	1.074	479					

a. R Squared = 0.718 (Adjusted R Squared = 0.703)

Qst.

Tests of Between-Subjects Effects

Dependent Variable:

Source	Type III Sum of Squares	df	Mean Square	F	Sig.	Partial Eta Squared	% Eta
Θ	3.356	1	3.356	170.369	0.000	0.277	0.22
L	2.625	1	2.625	133.234	0.000	0.230	0.18
σ_a	0.092	1	0.092	4.694	0.031	0.010	0.01
σ_s	8.253	2	4.126	209.445	0.000	0.485	0.38
Θ * L	1.205	1	1.205	61.166	0.000	0.121	0.09
Θ * σ_a	0.043	1	0.043	2.170	0.141	0.005	0.00
Θ * σ_s	5.747	2	2.874	145.856	0.000	0.396	0.31
L * σ_a	0.056	1	0.056	2.838	0.093	0.006	0.00
L * σ_s	5.284	2	2.642	134.090	0.000	0.376	0.30
σ_a * σ_s	0.164	2	0.082	4.169	0.016	0.018	0.01
Θ * L * σ_a	0.106	1	0.106	5.359	0.021	0.012	0.01
Θ * L * σ_s	2.429	2	1.214	61.645	0.000	0.217	0.17
Θ * σ_a * σ_s	0.094	2	0.047	2.397	0.092	0.011	0.01
L * σ_a * σ_s	0.086	2	0.043	2.173	0.115	0.010	0.01
Θ * L * σ_a * σ_s	0.204	2	0.102	5.190	0.006	0.023	0.02
Error	8.767	445	0.020				
Total	377.750	469					
Corrected Total	35.715	468					

a. R Squared = 0.755 (Adjusted R Squared = 0.742)
